# Direct Detection of Feline Coronavirus by Three Rapid Antigen Immunochromatographic Tests and by Real-Time PCR in Cat Shelters

**DOI:** 10.3390/vetsci9020035

**Published:** 2022-01-18

**Authors:** Veronika Vojtkovská, Gabriela Lukešová, Eva Voslářová, Jarmila Konvalinová, Vladimír Večerek, Dana Lobová

**Affiliations:** 1 Department of Animal Protection and Welfare and Veterinary Public Health, Faculty of Veterinary Hygiene and Ecology, University of Veterinary Sciences Brno, Palackého tř. 1946/1, 612 42 Brno, Czech Republic; lukesovag@vfu.cz (G.L.); voslarovae@vfu.cz (E.V.); konvalinovaj@vfu.cz (J.K.); vecerekv@vfu.cz (V.V.); 2 Department of Infectious Diseases and Microbiology, Faculty of Veterinary Medicine, University of Veterinary Sciences Brno, Palackého tř. 1946/1, 612 42 Brno, Czech Republic; lobovad@vfu.cz

**Keywords:** shelter cat, feline coronavirus, rapid immunochromatographic test

## Abstract

The aim of this study was the direct detection of feline coronavirus by real-time PCR and by three different rapid immunochromatographic (RIM) tests detecting antigens in faecal samples of shelter cats. Based on sensitivity and specificity calculated for each of the RIM tests, the utility of RIM tests was compared. Seventy faecal samples originating from shelter cats housed in quarantine were examined. Out of 70 samples analyzed by real-time PCR, 44 (62.9%) were positive. Significantly more cats (*p* < 0.05) tested positive than negative. Neither age nor sex of the cats played a significant role (*p* > 0.05) in the shedding status of the virus. The sensitivity of the RIM tests was found to be at low (<35%; RIM tests A and C) to satisfactory level (>50%, RIM test B). The number of virus particles determined by real-time RT-PCR analysis did not significantly correlate with the results detected by any of the RIM tests (*p* > 0.05). The results of this study indicate that the use of rapid antigen RIM tests in routine screening of FCoV shedding status in shelter cats is limited due to the occurrence of a high number of false negative results.

## 1. Introduction

Feline coronavirus (FCoV) is a major problem in the cat population worldwide. Two biotypes are recognized, the avirulent enteric form (Feline Enteric Coronavirus (FECV)) and the virulent form, Feline Infectious Peritonitis Virus (FIPV), which causes the fatal feline infectious peritonitis (FIP) [1]. Although FCoV infection is very common in cats, particularly in those housed in groups or at high density (typically in catteries, shelters, and multi-cat households), FIP usually arises only sporadically [2]. FCoV is a large enveloped single-stranded RNA virus that belongs to the order *Nidovirales*, family *Coronaviridae*, subfamily *Orthocoronavirinae*, and genus *Alphacoronavirus 1.* The virus is transmitted by the oral-faecal route, via ingestion of the virus in faeces and via sharing litter trays [3]. Most FCoV-infected cats remain asymptomatic, however, approximately 1–12% of infected cats develop FIP [4,5,6,7]. An extremely high mortality rate is characteristic for FIP; a spontaneous recovery of infected animals is very unlikely [8]. The clinical signs are traditionally divided into those associated with vasculitis causing non-septic effusions and those associated with granulomatous reactions in localized tissues. However, in reality, a varying combination of symptoms may occur with an overlap between the wet and dry manifestations [3]. 

Coronavirus-specific antibodies are present in up to 90% of cats in catteries and up to 50% of cats in single-cat households [5]. Feral or stray cats were found to generally have a lower prevalence of infection than those kept in households as they likely live in lower population densities and bury faeces outdoors, i.e., they are less exposed to contaminated faecal material in comparison with pet cats [9]. If the cat has not yet encountered the virus, placing it in a shelter poses a risk of infection, especially in the case of group housing. Stress plays an important role in FIP development; stressors such as moving to a new environment or cat density may increase the risk of an individual animal developing FIP [10]. The length of time spent in the shelter also increases the risk of exposure; cats housed in shelters longer than 60 days were found to be over five times more likely seropositive [11].

The only reliable way to prevent the development of FIP is to prevent FCoV infection in animals that have not yet encountered the virus. Based on the screening for the presence of antibodies and antigens on admission at the shelter, it is possible to separate seropositive individuals from negative ones [12] or to divide animals according to the virus shedding status. Nowadays, there are several options to detect FcoV antibodies and antigens. However, for shelter purposes, the method should be fast, simple, reliable, and affordable. Rapid immunochromatographic (RIM) tests were introduced as a diagnostic method meeting these criteria. The main substrate of these lateral flow tests is a nitrocellulose membrane in which a protein (an antigen or an antibody) is immobilized to capture gold conjugated detector antibodies/antigens. These techniques generate a qualitative result and enable a direct observation with the naked eye [13]. Invasively (whole blood, plasma, serum or effusion of the cat) but also non-invasively collected samples (faeces) can be used to detect FCoV antibodies or antigens in RIM tests.

The main objective of this study was the direct detection of feline coronavirus by three different RIM tests (detecting antigen in non-invasively taken faecal samples) and by the real-time RT-PCR in cat shelters. The hypothesis that the sensitivity and specificity of the tests would be higher than 50% was tested. Based on the determination of sensitivity and specificity, the utility of RIM tests in cat shelters was assessed.

## 2. Materials and Methods

### 2.1. Animals and Sampling

For the purposes of the sample collection, a cooperation with three private open-intake shelters located in the district of Moravia in the Czech Republic was established. Seventy shelter cats were included in the study. The group was composed of 36 males and 34 females aged from 2 months to 12 years (median age of 24 months). The approximate age of each animal was determined on its admission by the shelter staff. In order to determine whether age of cat played a role in FCoV shedding, cats were divided into four groups (x ≤ 6 months, 6 < x ≤ 12 months, 1 < x ≤ 8 years, x > 8 years; x = age of an animal). 

One faecal sample was taken from each cat 10 days after its admission to the shelter by the caretaker. The cats were quarantined at the time of sampling to prevent contact between the animals and thus to prevent an infection of potentially non-infected cats. The shedding status of the cats or information on the previous infection was not known at the time of sampling, as admitted cats were stray or abandoned animals. Each sample was assigned a score of 1 to 3 according to its consistency (1—firm, well-formed stool, 2—mostly formed but a softer stool, 3—mostly unformed, watery stool). Immediately after collection, samples were placed in the cooling box (4–8 °C), transported to the laboratory, and then stored in a freezer at a minimum of −20 °C.

### 2.2. RNA Isolation and Reverse Transcription

A commercial extraction kit the NucleoSpin^®^ RNA Stool (Macherey Nagel, Düren, Germany) was used to extract total RNA from 150 mg of each defrosted faecal sample. Extraction was conducted according to the manufacturer’s instructions. Quantity of extracted RNA was not measured by a spectrophotometric analysis as the sensitivity of the extraction kit used was declared by the manufacturer (the typical yield declared by the manufacturer was 10–30 µg RNA). Reliability of the RNA isolation was confirmed by using positive virus samples from the cell line. After extraction, total RNA was stored in a freezer at a minimum of −80 °C. Immediately after extraction, 5 µL was transcribed into cDNA using a commercial Transcriptor First Strand cDNA Synthesis Kit (Roche, Basel, Switzerland) with Random oligonucleotides. Two microliters of cDNA was used for PCR analysis.

### 2.3. Real-Time PCR—Identification of FCoV

The preparation of the quantitation standard was performed as previously described by Bubenikova et al. [14]. Following the manufacturer’s instructions, Xceed qPCR SG 1step 2 × Mix Lo-ROX (Institute of Applied Biotechnologies, Prague, Czech Republic) and a LightCycler^®^ 480 II (Roche, Basel, Switzerland) were used for detection and quantification of RNA P204 and P276 primers. Amplification conditions were as described by Bubenikova et al. [14]—initial denaturation performed 7 min at 95 °C was followed by 45 cycles of denaturation at 95 °C (10 s) and primer annealing/extension at 60 °C (30 s). The melting curve was created by raising the incubation temperature from 55 to 95 °C to confirm the specificity of the products obtained. The Samples whose melting temperature matched the control Tm (80 °C) were considered as positive. 10^5^ copies of the FCoV plasmid DNA were used as a positive control. As a negative control, PCR water was used. The specificity and sensitivity of the reactions calculated according to standard protocols were 1.0 and 0.958, respectively, with the limit of detection set as 10^2^ copies in 1 μL; the predictive values were 1.0 for a positive result and 0.909 for negative reactions. Based on the calibration curve, estimates of the numbers of virus particles generated by the Light Cycler were recorded and used for semi-quantitative phenotyping [14]. Threshold cycle (CT) number was used as the measure of viral load. The lower the CT, the more virus present in the sample. Samples with no signal at CT 30 were considered negative.

### 2.4. RIM Tests

Immediately after taking 150 mg of defrosted faeces for real-time PCR analysis, the rest of each sample was examined by three commercially available RIM tests to detect FCoV antigens (with the anti-FCoV monoclonal antibodies on the test line). The RIM tests originated from three different manufacturers (hereinafter referred to as A, B, and C as consent to publish the name of products was not obtained). All three RIM tests were performed under the same laboratory conditions and following the manufacturers’ instructions. The principle of using all three tests was same:

1. taking a small amount of faecal sample (in the case of tests A and C, the disposable swab was inserted into faeces to take the required amount; in the case of test B, a small plastic applicator was used to take a sufficient amount of sample),
2. inserting and mixing the faecal sample with assay diluent in the diluent tube,
3. placing the tube with the sample on a flat horizontal surface for sedimentation of gross faecal particles,
4. application of the supernatant on the test device (applying a few drops of supernatant into the sample well of a cassette in the case of tests A and B; placing the dipstick vertically into the sample tube in the case of RIM test C),
5. reading the test result visually—a correct test procedure was indicated by the appearance of a control line; if the line did not appear, the result was considered invalid; the appearance of a test line and a control line indicated the presence of FCoV antigen, if only a control line appeared in the result window, no FCoV antigen was present in the sample.

After performing the test, to confirm the result, each test device was checked independently by two evaluators. In agreement with the manufacturers’ instructions, even in case of occurrence of a weakly visible test line, the result was considered positive.

### 2.5. Determination of Sensitivity and Specificity of RIM Tests

Sensitivity and specificity were calculated for each of the RIM tests. To calculate sensitivity, the following equation was used:

$$Sensitivity\;\left(\%\right)=\frac{TP\times 100}{TP+FN}$$

*TP*: true positive—correctly identified as positive; *FN*: false negative.

For specificity calculation, the following equation was used:

$$Specificity\;\left(\%\right)=\frac{TN\times 100}{TN+FP}$$

*TN*: true negative—correctly identified as negative; *FP*: false positive.

### 2.6. Statistical Analysis

Statistical analysis of obtained data was performed in the statistical program Unistat 6.5 for Excel (Unistat Ltd., London, UK). A value of *p* ≤ 0.05 was considered statistically significant. The differences in the numbers of samples identified as positive and as negative by real-time PCR and by the RIM tests were analysed by the χ^2^ test (2 × 2 contingency tables). The χ^2^ test was also used to determine the differences between the numbers of samples correctly identified as positive and negative by RIM tests, to determine the differences between the numbers of positive and negative samples in individual sex and age categories, and to determine the differences in the numbers of samples in individual scores of faecal consistency. Differences in samples incorrectly identified as positive among RIM tests were analysed by the Fisher’s exact test. The Spearman’s rank correlation coefficient was used to verify the relation between the scores of faecal consistency and the cats’ virus shedding status determined by real-time PCR. The Spearman’s rank correlation coefficient was also used to verify the relation between the results given by individual RIM tests, between the results given by RIM tests and scores of faecal consistency, and between the number of virus particles calculated by LightCycler^®^ 480 II (Roche, Basel, Switzerland) and results given by individual RIM tests.

## 3. Results

### 3.1. Real-Time PCR Analysis, Faecal Consistency

Out of 70 samples examined by real-time PCR, 44 (62.9%) were positive. Significantly more cats (*p* < 0.05) were tested positive than negative. Neither age category (*p* > 0.05) (Table 1) nor sex of the cats (Table 2) played a significant role (*p* > 0.05) in the shedding status of the virus. 

In terms of faecal consistency, a score of 1 (firm, well-formed stool) was assigned to 35 (50%) of samples; 22 (31.4%) of stool samples were rated as mostly formed but softer (score of 2); 13 stool samples (18.6%) were rated a score of 3 (mostly unformed, watery stool). Significantly more samples (*p* < 0.05) were rated with a score of 1 than a score of 2 or 3. Scores of faecal consistency did not correlate with the cats’ virus shedding status (r_s_ = −0.0265, *p* > 0.05). The shedding of FCoV did not differ (*p* > 0.05) between cats with mostly unformed, watery stool (score of 3); cats with mostly formed but a softer stool (score of 2); and cats whose faecal consistency was normal (firm, well-formed stool, score of 1) (Table 3).

### 3.2. RIM Tests 

The characteristics of the used RIM tests are given in Table 4.

The number of positively tested samples (Table 5) differed significantly among RIM tests (*p* < 0.05). The highest number of positively tested samples was found by RIM test B, the lowest one by RIM test A.

The results given by RIM tests A and B did not correlate significantly (*p* > 0.05, r_s_ = 0.2025). Moderate strength of correlation was found between results given by RIM test B and C (*p* < 0.05, r_s_ = 0.5504) and by RIM test A and C (*p* < 0.05, r_s_ = 0.4792). 

The results of none of the tests significantly correlated with the faecal consistency score (Table 6).

### 3.3. Comparison of Results Given by RIM Tests and by Real-Time PCR

The results of real-time PCR analysis and RIM tests were compared (Table 7). The detected sensitivity of RIM test A and C was low (<35%); the sensitivity of RIM test B was at a satisfactory level (>50%). The specificity of the tests was at high (>90%, RIM test A, C) or at least at a satisfactory level (>50%, RIM test B). 

The total number of samples identified correctly did not differ significantly (*p* > 0.05) among RIM tests. Similarly, no significant difference was found in the number of incorrectly identified samples (*p* > 0.05). A significant difference was found among tests when comparing the numbers of samples correctly identified as positive (*p* < 0.05). The RIM test B identified as positive most samples that were also labelled as positive by PCR. The number of samples correctly identified as negative did not differ significantly (*p* > 0.05) among RIM tests. 

The numbers of false positive and false negative samples differed significantly among tests (*p* < 0.05). The majority of false results were found by RIM test A (up to half of all samples were incorrectly identified by this test). 

The number of virus particles calculated by the Light Cycler did not significantly correlate with the results detected by any of the RIM tests (Table 8). Thus, an increased number of viral particles in the sample did not increase the chance of antigen detection by RIM tests.

## 4. Discussion

The present study aimed to analyse faecal samples for the presence of FCoV by real-time PCR and three different RIM tests. The samples originated from cats quarantined after being admitted to a shelter. A comparison of three RIM tests was performed in terms of their ability to detect antigens and the overall utility in the cat shelter environment. To determine sensitivity and specificity, the results of the RIM tests were compared with the results obtained by the real-time PCR.

In multicat environments such as shelters, the detection of animals shedding virus is of a great importance, as it enables to separate shedders from non-shedding individuals [15]. In our study, analysis of faecal samples by real-time PCR revealed positivity in 44 samples, i.e., 62.9% of the total number of analysed samples. The number of cats shedding FCoV in their faeces found in our study can be considered relatively high since they were all stray and abandoned cats housed in quarantine after being placed in a shelter. In the previous studies, the prevalence of infection in this category of cats was reported to be low compared to pet cats [16] as cats with outdoor access have an opportunity to bury their faeces in distinct places and therefore have little to no contact with contaminated faeces [9,17,18]. The number of stray cats shedding FCoV ranged from 33% to 58% [19,20,21]. 

Sabshin et al. [20] reported a higher incidence of FCoV in cats having diarrhoea (58%) than in animals whose faecal consistency was normal (36%). However, their finding does not correspond with our results as we found no increase in FCoV shedding in cats with softer or watery stool. In shelter cats, faecal consistency may not always result from FCoV infection, it may be just a temporary response to stress from a new environment or a diet change. The duration of altered faecal consistency is individual for each cat.

The higher risk of shedding FCoV has also been linked with the lower age of cats [11,21,22,23,24]. The higher frequency of shedding in kittens is likely due to their immune system not being fully developed and allowing the virus to replicate efficiently [17,23]. Pedersen et al. [21] found as many as 90% of kittens and young cats up to 56 weeks of age to be shedding FCoV on admission to a shelter. In contrast with previous findings, age of cats was not associated with an increased shedding of FCoV in our study.

According to Pedersen et al. [21], regardless of age, FCoV excretion was found in 33% of cats on admission to the shelter. However, up to 60% of cats excreted the virus after 1 week. The results of our study are consistent with their finding, as we found 62.9% of cats shedding FCoV after 10 days spent in the shelter. However, in our study, all cats were housed individually for the first 10 days in the shelter to prevent contacts with other cats. Pedersen et al. [21] did not specify a housing system of cats in the shelter from which the samples were obtained in their study. An increase in FCoV shedding from 33% to 60% during 1 week was probably a result of several factors including the system of housing allowing a mutual contact between animals. In addition, stress, a factor potentially increasing the risk of FCoV shedding [23], probably also played a role.

Although the PCR method is considered adequate for detecting FCoV in faeces [25], it can be costly and time consuming, and thus, not ideal for routine screening of cats in shelters. An alternative to PCR is the use of commercially available RIM tests that are fast and easy to use. In addition, non-invasive faecal sampling is advantageous over RIM tests detecting FCoV antibodies in whole blood, serum, plasma, or effusion samples. Since the sensitivity and specificity of commercially available RIM assays detecting anti-FCoV antibodies were reported to be medium to high (sensitivity: 64.1–92.4%, specificity: 100%) by Addie et al. [12], sensitivity and specificity at a satisfactory level were also expected in RIM tests detecting antigens.

The results obtained by real-time PCR analysis in this study were compared with the results of RIM tests to determine their actual sensitivity and specificity. For RIM tests A and B, the manufacturers declared their sensitivity and specificity over 95% when compared with PCR. For the RIM test C, sensitivity and specificity versus PCR were unknown. Despite expectations, the hypothesis that the sensitivity of RIM tests would be higher than 50% was surprisingly refuted in two of the three RIM tests used; only RIM test B was found to have higher than 50% sensitivity (65.9%). Better results were found in specificity; all three RIM tests exceeded 70% (73.1–100%). The RIM test B had the highest sensitivity; however, this test also gave the highest number of false-positive results. As cats with an unknown medical history were included in our study, the possible explanation of the occurrence of false-positive results could be the cross-reactivity with other antigens present in faeces. According to the instruction sheet of one of the RIM tests, non-specific reactions may be caused by the presence of interfering endogenous or exogenous substances in the faeces, e.g., proteases, components of the intestinal mucosa, blood, grass, and cat litter. Faecal pH and viscosity may be other factors potentially affecting the test result according to the manufacturer’s instructions. Sample viscosity as a contributing factor to the increased risk of false-positive results in RIM tests has also been reported for SARS-CoV-2 antigen detection [26] and MERS-CoV [27].

Visible cat litter particles have been removed from faecal samples before testing them by RIM tests and before RNA isolation. In the case of unformed, watery faeces from the surface of which the cat litter was not possible to remove completely, a small amount of sample was taken from the inner part of the faeces using a disposable swab in order to avoid contamination by the litter as much as possible. Although it would be possible to avoid contamination by a direct collection of faeces from the cat’s rectum using a disposable swab, samples for analysis were taken from the cat’s toilets to simulate non-invasive sampling more likely to occur in the shelter in practice. Furthermore, RIM test B did not allow the collection of faeces from the rectum of cats; it was required to take a bigger amount of sample using a plastic applicator. All RIM tests used in the study allowed samples to be taken directly from faeces—the manufacturers’ instruction sheets did not recommend the sampling from the rectum over taking sample directly from the faeces. Therefore, the two procedures were considered equal.

Although the risk of non-specific reactions is likely to increase with the bigger sample amount (which was mainly the case of the RIM test B), on the other hand, the required larger amount of sample may be advantageous when considering inhomogeneous or nest-like dissemination of antigens in the faeces. Thus, taking a larger sample is also likely to increase the chance of an antigen being detected. In addition, the instruction sheet of the RIM test B recommends to homogenize the entire faecal sample before taking a small part of it for analysis, which should increase the chance of antigen capture. Sample homogenization was not mentioned in the instruction sheets of the RIM tests A and C, thus it was not performed for these tests. The large number of false-negative results obtained by RIM tests A and C might be related to the nature of the required sample and collection procedure (insertion of the disposable swab into one place of the faeces and collection of a small amount).

The results of our study did not prove a relationship between faecal consistency and RIM test results. Samples that were of a watery consistency did not tend to give positive results and vice versa. However, the consistency of faeces is likely to affect the outcome. RIM test B was the only test taking this factor into account in the testing procedure. RIM test B guidelines recommended to analyse two to three times larger faecal sample in case of its watery consistency compared to the amount of firm, well-formed faeces needed. However, no support for the assumption that watery faeces likely contain a lower concentration of antigen has been found in the scientific literature. The low detection limit could potentially explain the number of false-negative results provided by RIM tests, but as in the case of faecal consistency, the results given by RIM tests were not related to the number of viral particles detected by real-time PCR. The tests gave positive results even in the case of a small number of virus particles being detected by real-time PCR and negative results in the case of a presence of many virus particles in faeces. Contamination of negative samples by positive ones in the laboratory could potentially explain the number of false-negative results obtained by RIM tests. However, this scenario seems to be unlikely as extensive quality controls were included in the PCR protocol. 

The samples used for testing by RIM tests were frozen to a temperature of at least −20 °C after sampling in accordance with the manufacturers’ guidelines given in the instruction sheets. Due to the occurrence of a high number of false-negative results in RIM test A in our study, efforts were made to minimize the factors that could potentially affect the results. Although the manufacturer’s instruction sheet of the RIM test A declared that the sample could be permanently stored at a temperature of −20 °C, a simple experiment testing the effect of this type of storage on the results was performed. In the experiment, 10 randomly selected fresh faeces were taken from litter trays and tested directly in the shelter by RIM test A. After performing the tests, samples were transported to the laboratory and subsequently analysed by real-time PCR. Out of 10 samples, 6 samples were tested positive. However, all 10 samples were tested negative by the RIM test A.

From the perspective of functionality and utility of tests in shelters, testing is required to be cost-effective; quality of testing devices and clarity of instructions are also important. The largest number of RIM tests that had to be replaced with new ones due to poor quality of testing devices (damaged testing cassette, missing swab) or invalid test results (non-visibility of the control line in a test window) was found in RIM test C. This test also had the least elaborate instructions, e.g., it was not clear how long it is necessary to let the gross faecal particles sediment after mixing the sample with the diluent. Although the samples were allowed to sediment for at least 4 min, it was not possible to perform the test after this time either; after dropping the supernatant into the well of the test cassette, the fluid did not penetrate the test window with the test and control line. The test was performed correctly only after the application of the supernatant of the centrifuged sample into the well, which can be considered a disadvantage, as the shelters would have to be equipped with a laboratory centrifuge to perform this test. The duration of the whole test procedure did not differ significantly between the RIM tests; the result of each of the tests was obtained within 20 min (calculated from the beginning of the testing procedure to the moment of appearance of the result; in the case of a negative result, the moment the manufacturer determined as the last possible to read the result). 

## 5. Conclusions

Due to the overall low sensitivity of antigenic RIM assays found in this study, they cannot be considered a reliable method for detect shedding FCoV in the faeces of shelter cats. For routine testing, several factors should be considered when choosing a test, one of them being the reason for testing. If the shelter operators aim to separate animals that have already encountered the infection and animals that have not yet been infected, a suitable and cheap alternative to indirect immunofluorescent antibody tests (IFAT) or ELISA is the use of RIM tests detecting FCoV antibodies. The sensitivity of these tests has been reported to be very good [12]. The testing procedure of these tests is simple to perform, test kits are available commercially and provide results within a few minutes. However, the limitation of these tests is that even animals without antibodies can shed the virus [28,29]. These individuals should therefore be further tested for the presence of FCoV in faeces. For these purposes, the RIM tests detecting antigens that were subjects of this study would be beneficial. However, based on the findings of this study, more reliable results can only be obtained by using PCR. It is also important to note that the antigen RIM tests should not be used as a reliable alternative for antibody detection methods. Although it has been found that there is a relation between antibody titers and virus shedding [23,29,30], animals with high antibody titers but not shedding virus have been reported [29]. 

In a situation where it is primarily a matter of separating the cat shedding FCoV from non-shedders regardless of the presence of antibodies, the use of PCR instead of antigen tests should be the first choice. 

## Figures and Tables

**Table 1:** Shedding status of cats in individual age categories.

Age Category	Classification of Result	
	Positive (%)	Negative (%)
≤6 months	6 (60)	4 (40)
6 < x ≤ 12 months	7 (43.8)	9 (56.2)
1 < x ≤ 8 years	27 (71.1)	11 (28.9)
x > 8 years	4 (66.7)	2 (33.3)

**Table 2:** Shedding status of cats in individual sex categories.

Sex	Classification of Result	
	Positive (%)	Negative (%)
female	23 (67.6)	11 (32.4)
male	23 (63.9)	13 (36.1)

**Table 3:** Samples examined by real-time PCR in categories of faecal consistency.

Faecal Consistency Score	Classification of Result	
	Positive (%)	Negative (%)
1—firm, well-formed stool	22 (62.9)	13 (37.1)
2—mostly formed but a softer stool	15 (68.2)	7 (31.8)
3—mostly unformed, watery stool	7 (53.8)	6 (46.2)

**Table 4:** Specification of the individual RIM test.

	Antibody	Sensitivity Stated by the Manufacturer	Specificity Stated by the Manufacturer	Kit Storage	Sample Storage	The Total Duration of the Test Procedure	Number of Invalid Attempts (a New Test Device Had to Be Used)	The Test Requires the Use of Additional Equipment That Is Not Included in the Kit
RIM test A	anti-FCoV monoclonal antibody	>95% vs. PCR	>95% vs. PCR	15–30 °C	fresh faeces; 2–8 °C for max. 24 h; longer storage at −20 °C or below	approx. 15 min	1	no
RIM test B	anti-FCoV monoclonal antibody	>95% vs. PCR	>95% vs. PCR	15–25 °C	fresh faeces; 2–8 °C for max. 4 days; longer storage at −20 °C or below	approx. 20 min	3	no
RIM test C	anti-FCoV monoclonal antibody	>90% vs. ELISA, % vs. PCR unknown	>95% vs. ELISA, % vs. PCR unknown	2–30 °C	fresh faeces; 2–8 °C for max. 24 h; longer storage at −20 °C or below	approx. 15 min	6	Yes (use of centrifuge is needed)

**Table 5:** RIM tests results—positively and negatively tested samples.

RIM Test	Classification of Result	
	Positive (%)	Negative (%)
RIM test A	9 (12.9)	61 (87.1)
RIM test B	36 (51.42)	34 (48.6)
RIM test C	17 (24.3)	53 (75.7)

**Table 6:** Correlations between results of RIM tests and scores of faecal consistencies.

RIM Test	Faecal Consistency Score	
	Spearman’s Rank Correlation Coefficient	*p*-Value
RIM test A	−0.1662	0.1692
RIM test B	−0.1878	0.1195
RIM test C	−0.1964	0.1033

**Table 7:** Numbers of samples identified correctly and incorrectly by RIM tests, sensitivity, and specificity of RIM tests.

	RIM Test A (%)	RIM Test B (%)	RIM Test C (%)
the total number of samples identified correctly (out of 70)	35 (50.0)	48 (68.6)	39 (55.7)
number of samples correctly identified as positive	9 (12.9)	29 (41.4)	15 (21.4)
number of samples correctly identified as negative	26 (37.1)	19 (27.2)	24 (34.3)
the total number of samples identified incorrectly (out of 70)	35 (50.0)	22 (31.4)	31 (44.3)
number of samples incorrectly identified as positive–false positive samples	0 (0)	7 (10.0)	2 (2.9)
number of samples incorrectly identified as negative–false negative samples	35 (50.0)	15 (21.4)	29 (41.4)
sensitivity	20.5	65.9	34.1
specificity	100	73.1	92.3

**Table 8:** Correlations between results of RIM tests and virus particles calculated by Light Cycler.

RIM Test	Number of Virus Particles	
	Spearman’s Rank Correlation Coefficient	*p*-Value
RIM test A	0.2334	0.0536
RIM test B	0.2105	0.0825
RIM test C	0.1773	0.1450

## Data Availability

The data presented in this study are available on request from the corresponding author.
